# Annotations

**Published:** 1907-02-16

**Authors:** 


### ANNOTATIONS.

### The Effect of a Warm Climate on the Liver.

In a lecture delivered at the Polyclinic, Mr. James Cantlie expresses the opinion that too much responsibility is placed on the liver as the cause of various disturbances set up by the influence of tropical climates. At the same time it has to be recognised that such climates do exercise a very distinct influence on the hepatic functions. At first this influence is in the direction of physiological excitement—the liver is found to be increased in size and the hepatic activity is also increased, as is shown by the appearance in the stools of considerable quantities of bile. After some four to eight months the liver decreases in bulk, and soon is found to be actually less than normal; and with this the fæces become pale, the bowels constipated, and dyspeptic troubles appear. Now, one of two courses is possible. Either the liver remains small as a typical healthy tropical liver, or it increases in size, and can be recognised on palpation of the abdomen. The former is the normal condition of the liver in the tropics, whilst the enlargement just described is the result of want of exercise, associated very frequently with excess in food or drink. As seen in this country in persons who have been resident in the tropics, it is the small rather than the large liver which is associated with such intestinal disturbances as chronic diarrhœa or sprue. The influence of malaria on the liver is rather a vexed question, but this at least may be said, that an enlargement of the liver unaccompanied by a corresponding change in the spleen cannot be due to malaria. Further, many hepatic disturbances attributed popularly to the " deadly climate " are really due to the excessive use of alcohol.

### Municipal Bacteriologists.

The close and penetrating relationship between bacteriology and the numerous large questions which concern the public health is nowadays a familiar commonplace even to the junior medical student. The rapidity with which this position has been established may be gathered from the fact that when the Sale of Foods and Drugs Act became law in 1875, hardly a suspicion existed that the most serious defects in foods and drugs, as supplied to the public, might be due to the presence of micro-organisms. Hence has arisen a very curious position, as pointed out by Dr. E. C. Bousfield in a recent letter to the *Times*. The provisions of the Act can only be set in motion on the certificate of a " public analyst "; that is, an incriminated article can be condemned, and the vendor summoned to answer for the sale of a substance not of the nature and quality demanded by the purchaser, only when an official appointed as " public analyst " by the local authority has issued a formal report. Now " public analysts," whatever be their virtues, can, at least in the majority of cases, hardly claim to be expert bacteriologists. Therefore they are not usually in a position to condemn, say, a food substance on the ground of bacterial contamination. Take as a concrete example the presence of tubercle bacilli in milk. The recent Report of the Royal Commission on Tuberculosis will doubtless lead to the careful investigation of milk in this particular direction, and certainly it must be held that milk so contaminated is sold to the prejudice of the purchaser. Yet there may well be difficulty in providing the exact evidence which the law demands as necessary to secure a conviction. Manifestly there must be some modification of this position. The necessary reform appears to be the legal recognition of the expert bacteriologist as an official on whose verdict the local health authorities and the magistrates may take action. Some municipalities have already recognised the value of such an officer; and, indeed, no public health organisation can be considered complete without an appointment of this order.

### Babies in Bed.

In England about 2,000 children are every year suffocated through " overlaying," and 600 of these victims of parental carelessness die in London. In Germany such an accident is almost unknown. Dr. Wynn Westcott, in holding an inquest at Hackney on one of these unfortunate infants, remarked on this difference, and attributed it to the fact that in Germany a young child never sleeps in its parents' bed, but in a cot of its own. Here, at least among the poorer classes, the reverse is the rule. It cannot be denied that it is more convenient for the mother to have her baby in her own bed, especially when the child is breast-fed. But herein exactly lies the danger. A weary mother, falling asleep with the baby at her breast, is only too likely to fall over upon it, and the child, not having strength to pull itself away, is suffocated. The thing may happen without the mother being guilty of either drunkenness or criminal intent, though often enough there is ground for suspecting one or other of these factors. But it is so very rarely that either can be proved that the parent can almost certainly count upon escaping with nothing more than a warning to be more careful another time. The obvious remedy is to give the child a cot for itself, but it is doubtful if this will be adopted by the very persons for whom it would be most desirable; and there would be endless explanations about the risk of the child catching cold, or, if a little older, climbing out of the cot and falling on the floor, or in some other way coming to grief. And it must be admitted that when a baby has been accustomed to the warmth of its mother's bed, it does not take kindly to its own cot. To train the child to accept the change would involve one or two sleepless nights for the parents, and in a contest of this kind it is usually the smaller of the opponents who conquers. Perhaps before a change in practice became general it would be necessary to call in the aid of the Law. Law settles the question in Prussia, where it is illegal to have an infant under twelve months old in bed with its parents. It may be difficult to enforce a law like this, but when a child dies in its mother's bed the fact is regarded as a punishable offence, and the risk of punishment acts as a wholesome deterrent. But it is to be hoped that with the spread of health knowledge English mothers will do voluntarily what German ones do from fear.

